# Irisin Serum Levels in Metabolic Syndrome Patients Treated with Three Different Diets: A Post-Hoc Analysis from a Randomized Controlled Clinical Trial

**DOI:** 10.3390/nu10070844

**Published:** 2018-06-28

**Authors:** Alberto R. Osella, Graziana Colaianni, Mario Correale, Pasqua L. Pesole, Irene Bruno, Claudia Buongiorno, Valentina Deflorio, Carla M. Leone, Silvia Concetta Colucci, Maria Grano, Gianluigi Giannelli

**Affiliations:** 1 National Institute of Gastroenterology, “Saverio de Bellis” Research Hospital, Via Turi 27, 70013 Castellana Grotte, Italy; arosella@irccsdebellis.it (A.R.O.); mario.correale@irccsdebellis.it (M.C.); pesoleletizia@gmail.com (P.L.P.); irenebrunodiet@gmail.com (I.B.); buongiorno.claudia@gmail.com (C.B.); valentinadeflorio@yahoo.it (V.D.); carlaleone@interfree.it (C.M.L.); 2 Department of Emergency and Organ Transplantation, University of Bari, 70121 Bari, Italy; graziana.colaianni@libero.it (G.C.); maria.grano@uniba.it(M.G.); 3 Department of Basic Medical Science, Neuroscience and Sense Organs, University of Bari, 70121 Bari, Italy; silviaconcetta.colucci@uniba.it

**Keywords:** RCT, irisin, diet, metabolic syndrome

## Abstract

Background: Irisin, a hormone-like myokine, regulates energy homeostasis and mediates the benefits of physical activity on health. Methods: To estimate the effect of different diets on irisin concentrations in subjects with the Metabolic Syndrome (MetS). Methods: Subjects with MetS were derived from a population survey; 163 subjects were enrolled and randomized to a: Low Glycaemic Index (LGID), Mediterranean (MD) or Low Glycaemic Index Mediterranean (LGIMD) Diet, and the groups were compared, also with 80 controls without MetS. Sociodemographic, medical and nutritional data were collected and fasting blood samples drawn. Subjects underwent LUS and bioimpedentiometry. Generalized Estimating Equations were performed. Results: At baseline, lower irisin concentrations were observed in MetS subjects. Mean irisin levels increased in all diet groups but only the LGID group reached statistical significance, as well as showing an interaction between LGID and time at the sixth month examination (4.57, 95% CI −1.27, 7.87). There was a positive effect of Vegetable Proteins (0.03, 95% CI −0.01,0.06) and Saturated Fatty Acids (0.04, 95% CI 0.01, 0.07) on irisin concentrations. In the LGIMD, a positive effect on Fat-Free Mass (0.38, 95% CI 0.19, 0.57) and a negative effect on the Body Mass Index (−0.75, 95% CI −1.30, −0.19) were observed. Conclusions: There seems to be a link between diet and muscle physiology. We showed that patients following a LGID had higher levels of irisin, a promising biomarker of muscle activity.

## 1. Introduction

Metabolic syndrome (MetS) features a cluster of clinical conditions characterized by insulin resistance, resulting in a multifaceted clinical outcome [1,2]. MetS is becoming a major public health problem in Western countries; its prevalence increases with age and leads to a higher risk of developing cancer and chronic diseases [3,4,5,6]. The only known effective therapy for MetS is life style modifications, complying with recommendations to increase physical activity and keep to a healthy diet aimed at weight loss and improving the fat/fat-free mass ratio [7,8,9]. The Mediterranean diet has proven effective in patients with MetS [10], cardiovascular diseases [11], diabetes [12] and hypertension [13]. Multiple evidence has shown that the energy balance and improved metabolism are crucial in patients with MetS, as well as the type rather than the total amount of fat, the amount and type of protein intake, together with the glycaemic load and fiber content [14,15]. 

Although diet plays a crucial role in regulating the metabolic syndrome, it is not clear whether its composition influences the skeletal muscle distribution pattern. Skeletal muscle tissue is a preferential target of insulin since it is the primary site for insulin-induced utilization of glucose. Insulin resistance in the muscle is a metabolic alteration observed in obese and sedentary subjects [16]. 

In the past, it was shown that skeletal muscle is an endocrine organ that produces and secretes peptides, named myokines, whose synthesis is regulated by exercise [17]. Myokines act in several ways on several targets and have been recognized as candidates for the treatment of metabolic diseases owing to their ability to increase glucose uptake and stimulate lipolysis [18]. Irisin is a recently identified myokine produced by skeletal muscle during physical activity [18]. This myokine is released as a cleavage product of the transmembrane protein fibronectin type III domain-containing 5 (FNDC5), that is highly expressed under the control of peroxisome proliferator-activated receptor gamma coactivator 1-alpha (PGC1α) in mice and humans. When discovered, irisin was defined as the hormone triggering the so-called “browning response”, that is, the transdifferentiation of white adipocytes into the brown adipocytes involved in the thermogenesis process [19]. More recently, it has been shown that irisin displays an anabolic effect on bone and muscle tissues at much lower concentrations than those inducing a browning response, indicating that the skeleton could be this myokine’s first target [20,21]. Human studies have shown that physical inactivity, overweight and obesity can lead to a reduction in irisin serum concentrations [21,22]. 

The effects of a controlled diet on circulating irisin concentrations have been little investigated and no studies have investigated the impact of diet on the relationship between irisin and MetS. The aim of this study is, therefore, to explore the effect of different diets on irisin serum concentrations in patients with MetS.

## 2. Materials and Methods

### 2.1. Study Design

This study is registered at [23], Identifier: NCT02356952. 

MEDIDIET was a parallel-arms randomized controlled clinical trial. Subjects with MetS were drawn from the MICOL study conducted at the National Institute of Gastroenterology, “Saverio de Bellis” Research Hospital. MICOL was a population survey conducted in 2005–2006 to study relationships between diet and chronic diseases. In MICOL, a random population sample (aged 30–89 years) was drawn from the electoral roll of a small town of southern Italy; 2973 subjects were examined (response rate 70%), and 1042 had MetS according to the NCEP-ATP III criteria [22].

### 2.2. Participants Selection

Between December 2007 and April 2008, MICOL subjects who had been screened in 2005–2006 and had MetS (1042 subjects) were invited to undergo further examination: 556 subjects responded, and 163 of 387 subjects (100 males, 63 females) were still affected by MetS. We included subjects treated with statins, anti-hypertensives, oral antidiabetics, but excluded subjects in insulin treatment. Patients were requested not to change their exercise habits after enrollment in the study; 80 subjects from the same population random sample without MetS matched for age, gender and Body Mass Index (BMI) were selected as control group (no diet).

The trial was conducted in collaboration with General Practitioners, approved by the Ethics Committee of our Institution and, in accordance with the Helsinki Declaration, all participants provided written informed consent.

### 2.3. Randomization

Participants were randomly assigned by simple randomization procedures (computerized random numbers sequence) to one of three diets; a one-to-one ratio was used to allocate subjects.

Blinding was maintained by firstly assuring the staff and participants that each diet was based on healthy principles. Participants were followed for the duration of the trial and the dietitian was assigned on a daily random basis. Moreover, only one intervention group was called in each day and only one patient at each date, to reduce to a minimum the information exchange among participants. Staff members who assessed outcome were unaware of the diet assigned. Only one of two radiologists performed outcome measurements each day and this order was also randomly assigned. In the outcome measurements made at the third and sixth months, the radiologists were unaware of the previous measurements.

### 2.4. Baseline Examination

Initial screening included a complete medical history, physical examination, blood sampling. The brachial blood pressure at rest was always measured by a trained nurse, using a sphygmomanometer with an appropriate cuff. Blood samples were taken between 8:00 and 9:30 a.m. with participants fasting for at least 12 h. Anthropometric measurements (weight, height, waist circumference) were taken by three dietitians; the dietitians also administered a validated semi-quantitative food frequency questionnaire and carried out bio-impedentiometric analysis (BIA) (Akern SRL, Via Lisbona 32/34 50065 Pontassieve, Italy). 

### 2.5. Dietary Intervention

A dietary tool was created and tested to administer diets qualitatively based on the “traffic light” method, which divides foods into color-coded categories: green (foods that can be eaten freely), yellow (foods that can be eaten in moderation), red (foods that are prohibited).

We characterized the following diets: Mediterranean diet (MD), built using the Trichopoulou A. et al. study [24]; Low Glycaemic Index diet (LGID) based on the Elia A. study [25] and Low Glycaemic Index Mediterranean diet (LGIMD), created by integrating the Trichopolou A. et al. [25] and Elia A. studies [26], and adapting them to our population.

Individuals recorded what they ate on a daily diet diary. The main objectives pursued in the creation of the diets and the administration and monitoring tools were: (1) to let subjects choose their foods; (2) to help them monitor what they ate. The characteristics of the three diets are described in Appendix A and their main nutritional composition is described in Table S11. Furthermore, patients were asked to not change their exercise habits after enrollment in the study.

### 2.6. Outcomes

Primary outcome measures were MetS, MetS score and its components. Secondary outcomes were anthropometric and biochemical markers; Fat (FM) and Fat-Free Mass (FFM) and Non-Alcoholic Fatty Liver Disease (NAFLD) score (measured by Liver Ultrasound (LUS). Irisin serum concentrations were also considered as a secondary outcome. As irisin was discovered after the trial had been conducted, this is a post-hoc analysis. 

Irisin concentrations assessment was performed using a competitive Enzyme Linked-Immunosorbent Assay (ELISA) for the quantitative determination of irisin in human biological fluids (AdipoGen Life Sciences^®^, Adipogen Corporation, 9853 Pacific Heights Blvd., Suite L, San Diego, CA, USA) [27]. The irisin assessment was performed at baseline and the 3rd and 6th months of follow-up.

### 2.7. Implementation

Subjects were followed up monthly for dietary counseling, checking their diaries and controlling their anthropometric parameters. After 12 and 24 weeks from the beginning of the study the subjects again underwent blood sampling, BIA and anthropometric measurements. 

### 2.8. Statistical Analysis

The primary analysis was intention-to-treat in all participants. Cross-tabulations between interventions and socio-demographic, life-style and biological variables were performed to describe the participants.

Dietary Records were analyzed using MètaDieta^®^ software and the results expressed as percentage of total calorie intake for each food item consumed. To estimate the compliance with the prescribed diet the Mediterranean Adequacy Index (MAI) was used. Random week and week-end days were chosen from the second and fourth month of intervention. The MAI was estimated according to gender and month to clearly describe compliance. Compliance was defined as positive and expressed as percentages if the subject’s ratio of calories derived from foods of the LGID or MD or LGIMD versus foods not in the LGID or MD or LGIMD subject was equal to or above the median value.

A Generalized Estimating Equation was used to estimate the effect of the three diets, biochemical markers, food groups and nutrients on irisin concentrations, measured together and in each diet separately This type of model is useful in biomedical studies to estimate how the average outcome changes in response to correlated data. A gamma distribution (link identity) for the response was assumed and an unstructured correlation matrix was set to the data. Gender (categorical), BMI, HOMA-IR Test and age (continuous variables) were included as covariates. The results obtained were expressed on the natural scale as means ±95% Confidence Intervals (95% CI). As a post-estimation tool, marginal distribution of the response was implemented to probe the expected irisin values using several explanatory variables. Statistical analysis was carried out using Stata statistical software (version 15.1), StataCorp, 4905 Lakeway Drive, College Station, TX, USA.

## 3. Results

### 3.1. Sample Description

Participants’ characteristics are shown in Table 1. The MD group included 54 individuals, LGIMD 53, and LGID 56. Only four of 54 subjects in the MD group, 4 of 56 in the LGID group, and six of 53 in the LGIMD group had been lost to follow-up at the 24th week. 

Age class was homogeneously distributed among intervention groups, ranging from 11.1 to 29.6% for each age class-diet combination. About 40% were females and the mean age was 57.6 (11.8) (men 56.8 (12.0), women 58.8 (11.3)). All other characteristics were equally distributed among treated subjects (Table 1). Mean age by treatment was 54.9 years (13.9) among controls, 57.5 (10.7) for LGID, 59.4 (10.4) MD and 58.3 (9.8) for LGIMD. Although the BMI score was equally distributed among intervention groups, it was slightly lower among controls, without reaching statistical significance. 

Phase Angle, the cornerstone parameter of BIA, was high among all groups, reflecting a good nutritional status in this population. All other characteristics were equally distributed among treated subjects. As expected, the NAFLD prevalence ranged from 19.5 to 30.8% (Supplementary Materials Table S1).

### 3.2. Time and Compliance with the Diet Influences Serum Concentrations

**Figure 1:**
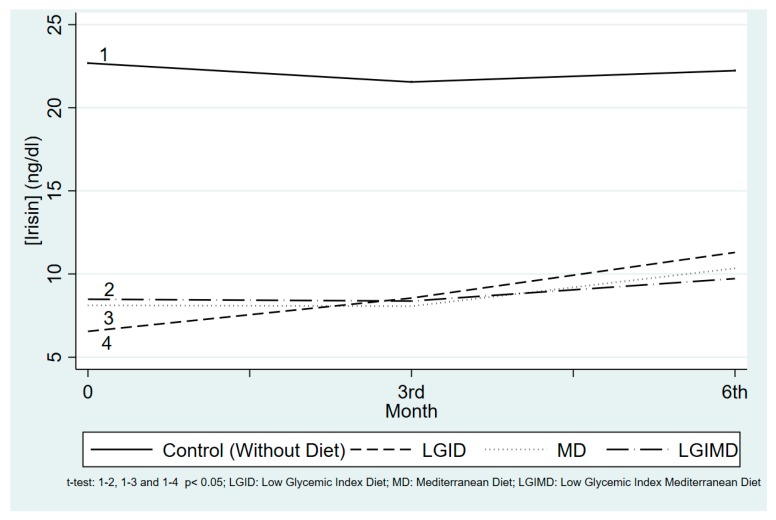
Observed irisin Concentrations by Diet and Time. Medidiet Trial, Castellana Grotte (Italy). *t*-test statistic.

As shown in Figure 1, at enrollment MetS subjects displayed lower irisin concentrations than controls. During follow-up, mean irisin concentrations tended to increase in all three groups but the difference became significant only in the LGID diet after six months.

Mean irisin concentrations by gender and month were evaluated to check compliance with the intervention diet, as shown in Table 2.

Overall compliance was higher among females and increased from the second to the fourth month. Compliance was about 58.9 for men and 62% for women. There was a high, constant compliance with LGID in the two periods considered among both men and women. Compliance with LGIMD was low (range 9–20%). Table 2 also shows mean irisin concentrations by compliance, month and Gender. There were statistically significant mean differences among men between the two periods considered in terms of non-compliant subjects in LGID, compliant and non-compliant subjects in MD and compliant subjects in LGIMD. Among women, there were statistically significant mean differences between compliant and non-compliant subjects for LGID and MD. 

### 3.3. Effects of Diets and Some Nutrients on Serum Irisin Concentrations

The GEE analysis results are presented in Table 3. As shown, the overall principal effect of each diet without considering the follow-up was to lower irisin levels by about 15% as compared with controls (*p* < 0.01). No main effect of time on irisin concentrations was found. There was a significant effect modification between LGID and time on irisin concentration at the sixth month of follow-up (4.57, 95% CI −1.27, 7.87). All these estimates are adjusted for demographics (Gender, Age) and metabolic characteristics (BMI, HOMA-IR test).

**Figure 2:**
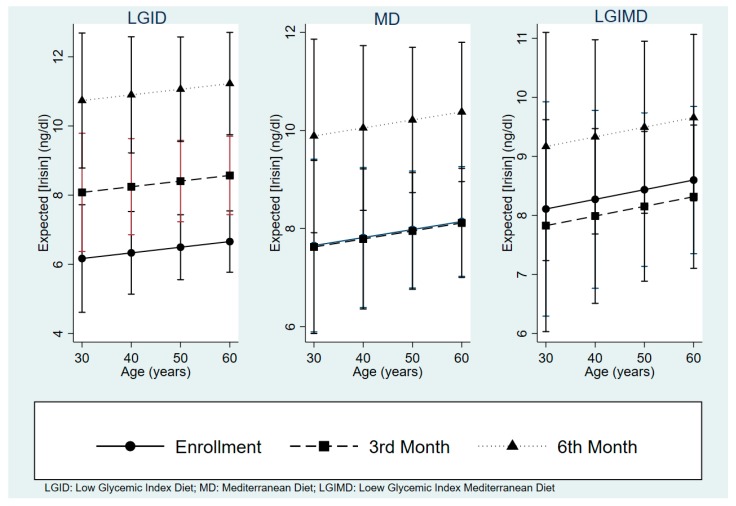
Expected irisin Concentrations by Diet, Age and Time. Medidiet Trial, Castellana Grotte (Italy). Generalized Estimation Equation Analysis.

Expected irisin concentrations are shown in Figure 2. There was a constant rise of irisin concentrations with LGID, which reached higher concentrations as compared with the other diets. MD and LGIMD not only did not reach the final LGID irisin concentrations but also the intermediate measurements were lower. There was also a slight trend to increase of irisin with age but did not reach statistical significance.

There was also a significant positive effect of Vegetable Proteins (0.03, 95% CI −0.01, 0.06) and Saturated Fatty Acids (0.04, 95% CI 0.01, 0.07) (Supplementary Materials Table S2). Expected irisin concentrations by Vegetable Protein Intake are shown in Supplementary Materials Figure S1. Irisin concentrations exhibit a steady rise with increased intakes of Vegetable Protein and Saturated Fatty Acids. Overall, there was a significant negative effect of GGT, and a minor but significant effect of Cheeses and Processed Meats on irisin concentrations. No effect of BIA parameters was observed (Supplementary Materials Figures S1–S3 and Tables S1–S4).

### 3.4. Each Diet’s Effect on Irisin Concentrations

When GEE models were applied to each intervention diet a positive effect on FFM (0.38, 95% CI 0.19, 0.57) of LGID was observed. Supplementary Materials Figure S2 shows the expected irisin concentration at different FFM percentages. A steady rise of irisin concentrations was observed with higher percentages of FFM body composition. On the contrary, a negative effect of the LGIMD BMI (−0.75, 95% CI −1.30, −0.19) on irisin concentrations was observed (Supplementary Materials Table S6 and Figure S3).

All results related to primary and secondary end-points are available as Supplementary Materials Tables S5–S11.

## 4. Discussion

In this study, we observed that irisin concentrations are significantly lower in subjects affected by MetS than in a general population random sample. When MetS patients were exposed to different types of diets, irisin concentrations increased significantly when compliance with LGID was high. These results highlight the effect of diet on irisin concentration when the nutritional intervention is sustained and controlled.

Irisin was initially described as a myokine that triggers the browning of white adipose tissue, thus increasing energy expenditure [18]. An anabolic effect was later shown on bone tissue in both physiological and pathological conditions [19,28]. Further observations demonstrated positive irisin effects on lipid and glucose metabolism [21] as well as on insulin secretion and sensitivity [29]. However, studies prevalently performed in humans have yielded controversial data regarding the association of irisin with obesity, insulin resistance, glucose disorders [30,31], fat mass and BMI [32,33,34,35].

Discrepancies have also been reported concerning irisin concentrations in MetS in adults [36,37] and prepuberal children [38] and adolescents [39]. Although the regulation of irisin in subjects with MetS remains controversial, the divergent findings across studies may be explained by differences in study populations. Patients selection and differences in dietary components, compliance and study duration can be critical in determining the different findings.

Here, we show that at enrollment, all subjects with MetS had lower concentrations of circulating irisin compared with the control group. After follow-up, we found a significant effect modification between LGID and time on irisin concentrations, suggesting that the effect of diet on irisin concentrations is strictly dependent on time. In patients on MD and LGIMD (lower diet compliance), intermediate irisin measurements were lower than the values for LGID and after six months, did not reach those detected in LGID, even if an upward trend was observed in both groups. These data further support the notion that the benefits of a diet are related to the composition of foods as well as to long term compliance.

So far, few studies have analyzed the impact of diets on irisin serum concentrations and results have been controversial. Irisin concentrations have been shown to be positively associated with fruit but negatively correlated to meat consumption [40], whereas in another study irisin was not affected by food intake [41]. It has also been shown that an increasing calorie intake is associated with lower irisin concentrations [42]. 

A randomized, controlled trial compared the effects of two different 2-month-long hypocaloric dietary interventions on irisin concentrations in patients with MetS. In particular, the control diet was based on the American Heart Association guidelines [43,44] and a similar decrease in irisin concentrations was observed in both dietary groups. In another study, a group of 94 obese patients was enrolled in a weight loss program based on an 8-week hypocaloric diet and weight maintenance follow-up. After intervention, irisin concentrations decreased in parallel with body weight reduction but returned to the baseline concentrations in those patients who regained the lost weight after 24 weeks [45].

Although these last two findings are not in agreement with our results, the different diet regimen, duration and differences in dietary components seem to be critical factors explaining the results. To our knowledge, our study is the first 6-month long randomized trial to monitor irisin serum concentrations, together with compliance to the diet. The greater the compliance the greater the irisin concentrations. Moreover, we observed a positive effect on Fat Free Mass of LGID and a negative effect on BMI of LGIMD. 

Our results also show that vegetable proteins increase, while cheeses and processed meats decrease, irisin concentrations. This is a remarkable result given the evidence of the different effect of vegetable and animal proteins on cardiovascular disease, since vegetarian people tend to have lower arterial pressure and plasma cholesterol than their omnivorous counterparts [46].

Some methodological issues need to be considered. Strengths of this study are the population-based nature of the study, the RCT design and the measurement of compliance with each diet. Furthermore, to better reflect the eating behavior of participants Dietary Patterns rather than foods were studied, outcome assessment was performed blinded and an intention-to-treat analysis was carried out. Limitations include the lack of measured physical activity as the request not to modify their habits during follow-up may not be sufficient, as well as the reliance on self-reported food intake. However, the high validity and reproducibility of the EPIC FFQ was demonstrated. There are also some drawbacks to post-hoc analysis. Post hoc analysis is fraught with confounders and so only hypotheses can be generated, therefore further research is needed to assess the effects of specific food groups on irisin levels [47].

Our study attributes a considerable value to the dietary habits of the Apulia region population, where the study was conducted and where foods with a high vegetable protein content are much preferred to those of animal origin.

## 5. Conclusions

In conclusion, this study shows that serum irisin concentrations increase in patients with Mets on a LGID only if the subjects maintain a proper compliance to the diet over time. This may suggest that factors such as a specific diet may be effective in modulating the synthesis of irisin concentration. Furthermore, irisin assessment as a marker could be useful in clinical practice to highlight and monitor a metabolic disorder.

## Figures and Tables

**Table 1:** Socio-demographic Characteristics of Participants.

	CONTROL		LGID		MD		LGIMD	
	No.	%	No.	%	No.	%	No.	%
Age (years)								
<40	16	72.7	2	9.1	2	9.1	2	9.1
40–49	20	44.4	13	28.9	7	15.6	5	11.1
50–59	13	18.3	19	26.8	18	25.4	21	29.6
60–69	20	33.3	14	23.3	15	25.0	11	18.3
70 or more	11	33.3	7	21.2	9	27.3	6	18.2
Gender								
Male	47	34.3	29	21.2	33	24.1	28	20.4
Female	33	35.0	26	28.0	18	19.4	17	18.3
Status								
Single	5	50.0	3	30.0	1	10.0	1	10.0
Married	72	37.9	43	22.6	40	21.1	35	18.4
Widowed	1	9.1	4	36.4	2	18.2	4	36.4
Divorced	2	66.7	0	0.0	0	0.0	1	33.3
Education								
Illiterate	1	16.7	2	33.3	3	50.0	0	0.0
Primary School	15	24.6	15	24.6	15	24.6	16	26.2
Middle School	26	38.2	17	25.0	18	26.5	7	10.3
High School	29	38.2	17	22.4	12	15.8	18	23.7
Graduate	4	26.7	4	26.7	3	20.0	4	26.7
No Information	5	100.0	0	0.0	0	0.0	0	0.0
Total	80	34.6	55	23.8	51	22.1	45	19.5

LGID: Low Glycaemic Index Diet; MD: Mediterranean Diet; LGIMD: Low Glycaemic Index Mediterrranean Diet.

**Table 2:** Compliance with the Low Glycaemic Index Mediterranean, Mediterranean and Low Glycaemic Index Diet, and Mean irisin level by Gender and Month.

		Second Month		Fourth Month	
		Compliance		Compliance	
**Male**	**Diet**	**No**	**Yes**	**No**	**Yes**
		***n* (%)**	***n* (%)**	***n* (%)**	***n* (%)**
		**Mean (±SD)**	**Mean (±SD)**	**Mean (±SD)**	**Mean (±SD)**
	LGID	44 (53)	39 (47)	34 (41)	49 (59)
	[Irisin] #	8.8 (0.6) *	7.9 (0.8)	10.8 (0.7) *	9.4 (1.1)
	MD	30 (41)	43 (59)	30 (41)	43 (59)
	[Irisin]	8.7 (0.6) *	8.5 (0.8) **	10.2 (0.7) *	10.5 (1.1) **
	LGIMD	75 (90)	8 (10)	75 (90)	8 (10)
	[Irisin]	7.5 (0.8)	6.9 (0.9) **	8.3 (0.7)	9.1 (0.7) **
**Female**	**Diet**	**No**	**Yes**	**No**	**Yes**
		***n* (%)**	***n* (%)**	***n* (%)**	***n* (%)**
		**Mean (±SD)**	**Mean (±SD)**	**Mean (±SD)**	**Mean (±SD)**
	LGID	27 (44)	35 (56)	16 (28)	41 (72)
	[Irisin]	7.6 (0.7) *	8.2 (0.8) **	11.6 (1.1) *	10.4 (0.8) **
	MD	20 (39)	32 (61)	19 (38.00)	31 (62.00)
	[Irisin]	7.6 (0.8) *	8.0 (0.7) **	11.2 (1.0) *	10.7 (0.9) **
	LGIMD	50 (81)	12 (19)	45 (79)	12 (21)
	[Irisin]	7.7 (1.2)	8.4 (0.9)	7.0 (1.0)	8.5 (0.6)

LGID: Low Glycaemic Index Diet; MD: Mediterranean Diet; LGIMD: Low Glycaemic Index. Mediterrranean Diet. # Mean irisin Level (ng/mL); * *p* < 0.05, ** *p* < 0.01.

**Table 3:** Overall Effect of Different Diets on irisin Levels MEDIDIET, Castellana Grotte, BA, Italy 2009.

Variable	Crude Estimates	Adjusted by Age, Gender, BMI, HOMA-IR
Control (Reference)	0.00	0.00
MD	−14.57 #	−15.77
LGID	−16.13 #	−14.29
LGIMD	−14.20 #	−13.82
3rd month	0.00	0.00
6th month	0.00	0.00
MD*Month(3rd)	−0.05	−0.03
MD* Month (6th)	2.22	2.24
LGID* Month (3rd)	2.00	1.91
LGID* Month (6th)	4.74 #	4.57 #
LGIMD* Month (3rd)	−0.11	−0.28
LGIMD* Month (6th)	1.24	1.06

CI: Confidence Interval; # *p* < 0.01; LGID: Low Glycaemic Index Diet, MD: Mediterranean Diet LIGMD: Low Glycaemic Index Mediterranean Diet.

## Supplementary Materials

The following are available online at http://www.mdpi.com/2072-6643/10/7/844/s1, Figure S1: Expected (lrisin)(ng/dL) by Vegetable Protein and Saturated Fatty Aciods Daily Intake, Figure S2: Expected (lrisin)(ng/dL) by Free Fat Mass (%) in LGID, Figure S3: Expected (lrisin)(ng/dL) by Body Mass Index in LGIMD, Table S1: Biochemical and Metabolic Characteristics of Participants, Table S2: Effect of Biochemical Markers on Irisin Levels. MEDIDIET, Castellana Grotte, BA, Italy 2009, Table S3: Effect of Food Groups on Irisin Levels MEDIDIET, Castellana Grotte, BA, Italy 2009, Table S4: Effect of BIA on Irisin Levels MEDIDIET, Castellana Grotte, BA, Italy 2009, Table S5: Descriptive statistics (mean ± standard deviation or relative frequency) of the main characteristics of the subjects with metabolic syndrome (MetS), randomized to Mediterranean diet (MD), low glycaemic index diet (LGID), low glycaemic index Mediterranean diet (LGIMD), Table S6: Mean and standard deviation (M ± SD) of the metabolic syndrome (MetS) score and its components at baseline (T0), and of their variation at 3 (Δ0–3) and 6 months (Δ0–6) in subjects with MetS randomized to Mediterranean diet (MD), low glycaemic index diet (LGID), and low glycaemic index Mediterranean diet (LGIMD), Table S7: Mean and standard deviation (M ± SD) of metabolic and anthropometric variables at baseline (T0), and of their variation at 3 (Δ0–3), and 6 months (Δ0–6) in subjects with metabolic syndrome (MetS) randomized to Mediterranean diet (MD), low glycaemic index diet (LGID), and low glycaemic index Mediterranean diet (LGIMD), Table S8: Analysis of variance for repeated measures, at baseline (T0), 3 (T3) and 6 (T6) months of the metabolic syndrome (MetS) score and its components in subjects with metabolic syndrome (MetS) randomized to Mediterranean Diet (MD), low glycaemic index diet (LGID), low glycaemic index Mediterranean diet (LGIMD), Table S9: Analysis of variance for repeated measures, at baseline (T0), 3 (T3) and 6 (T6) months of metabolic and anthropometric variables in subjects with metabolic syndrome (MetS) randomized to Mediterranean diet (MD), low glycaemic index diet (LGID), low glycaemic index Mediterranean diet (LGIMD), Table S10: Multiple linear regression models of Fat mass and Glycated Hemoglobin on Mediterranean diet (MD), low glycaemic index diet (LGID), and low glycaemic index +Mediterranean diet (LGIMD) at 3 and 6 months, controlling for age, gender, and baseline value of each variable (MD is the comparison diet), Table S11: Energy and nutrients intake in Mediterranean diet (MD), low glycaemic index diet (LGID), and low glycaemic index Mediterranean diet (LGIMD).

## Appendix A

### Appendix A.1. Composition of the Diets

#### Appendix A.1.1. Food to be Eaten Regularly

Mediterranean diet (MD)—Raw vegetables (lettuce, tomatoes, cucumbers, celery, carrots, radishes, etc.) steamed or boiled vegetables (beets, turnips, chicory, cauliflower, broccoli, etc.), fresh vegetables (peas, beans, green beans), pulses (lentils, chickpeas, beans, broad beans, soy), fish (anchovies, sardines, mackerel, etc.), cod, swordfish, fresh tuna, shellfish, canned tuna, bread, rice, pasta, tomato sauce, potatoes, extra virgin olive oil (raw) fresh fruits (apples, pears, oranges, grapefruit, kiwi, etc.) dried fruit (walnuts, almonds, dried figs: max 5 pieces), thick honey (pure), natural water, coffee (without sugar or artificial sweetener).

Low glycaemic index diet (LGID)—Raw vegetables (lettuce, tomatoes, cucumbers, celery, carrots, radishes, etc.) steamed or boiled vegetables (beets, turnips, chicory, cauliflower, broccoli, etc.) fresh vegetables on their own (peas, green beans), pulses alone (lentils, chickpeas, beans, broad beans, soy), whole wheat pasta with vegetables, whole wheat pasta with vegetables, brown rice with legumes, brown rice with vegetables, extra virgin olive oil (raw) fish, mollusks and crustaceans; canned tuna, unsweetened fresh fruit (apples, pears, oranges, grapefruit, kiwi, peaches, etc.) unsweetened nuts (walnuts, almonds: max 5 pieces), natural water, coffee (without sugar or with artificial sweetener).

Low glycaemic index Mediterranean diet (LGIMD)—Raw vegetables (lettuce, tomatoes, cucumbers, celery, carrots, radishes, etc.) steamed or boiled vegetables (beets, turnips, chicory, cauliflower, broccoli, etc.) just fresh vegetables (peas, beans, green beans), pulses alone (lentils, chickpeas, beans, broad beans, soybeans), whole wheat pasta with vegetables, whole wheat pasta with vegetables, brown rice with vegetables; brown rice with vegetables, extra virgin olive oil (raw), fish (anchovies, sardines, mackerel, etc.), cod, swordfish, fresh tuna, shellfish, canned tuna, unsweetened fresh fruit (apples, pears, oranges, grapefruit, kiwi, peaches, etc.), unsweetened nuts (walnuts, almonds: max 5 pieces), natural water, coffee (without sugar or artificial sweetener).

Foods to be Eaten in Moderation

MD—White meat (chicken, turkey, rabbit), milk and yogurt, dairy products (cheese, smoked cheese, ricotta) cheese (parmesan, pecorino cheese, etc.) eggs, dry biscuits and rusks, wine.

LGID—Whole wheat bread, whole wheat pasta with meat sauce; whole wheat pasta sauce, potatoes (read only), milk and yogurt, dairy products (cheese, smoked cheese, ricotta) cheese (parmesan, pecorino cheese, etc.) eggs, white meat (chicken, turkey, rabbit), red meat (beef, veal, pork, horse, etc.) salamis, sausages (raw or cooked ham, cured ham, salami, mortadella, etc.). sugary fruits (bananas, persimmons, grapes); pure thick honey; whole wheat biscuits, wine.

LGIMD—Whole wheat bread, whole wheat pasta with simple sauce, potatoes (read only), milk and yogurt, dairy products (cheese, smoked cheese, cottage cheese), cheese (parmesan, cheese, etc.) eggs, white meat (chicken, turkey, rabbit), sugary fruit (bananas, persimmons, grapes); pure thick honey, whole wheat biscuits, wine.

#### Appendix A.1.2. Foods to be Avoided Completely

MD—Red meat (beef, veal, pork, horse, etc.) Sauce, canned meat, sausages, cured meats (raw or cooked ham, dried beef, bacon, salami, mortadella, etc.); farmed fish, butter, margarine, mayonnaise, cream, pizza, french fries or baked-goods, crackers, pretzels, bread sticks, set them, cakes and breakfast cereals, sugar, jam, fruit syrup and candied chocolate candies, cakes, pastries, croissants, shortbread, biscuits, ice cream and ices; alcoholic beverages and spirits (brandy, grappa, liqueurs, whiskey) and non-carbonated soft drinks (orange juice, coca-cola, fruit juice, etc.) and beer.

LGID—Refined foods, white rice, white bread, pizza, french fries or baked goods, crackers, pretzels, bread sticks, bread and breakfast cereals, butter, margarine , mayonnaise, cream, sweet dried fruit (dried figs, dried dates, prunes, raisins), fruit in syrup and candied sugar, jams, candies and chocolates, cakes, sweets, pastries; croissants, pastries and cakes, shortbread, biscuits, dry biscuits, toast, ice cream and ices; alcoholic beverages and spirits (brandy, grappa, liqueurs, whiskey), non-alcoholic beverages and non-carbonated (orange juice, coca-cola, fruit juice, etc.) and beer.

LGIMD—Refined pasta, white rice, white bread, pizza, french fries or baked goods, crackers, pretzels, bread sticks, bread and breakfast cereals, butter, margarine, mayonnaise, cream, red meat (beef, veal, pork, horse, etc.) sauce, canned meat, sausages (raw or cooked ham, dried beef, bacon, salami, mortadella, etc.) farmed fish, sweet dried fruit (dried figs, dried dates, prunes, raisins), fruit in syrup and candied sugar, jams, candies and chocolates, cakes, sweets, pastries, croissants, pastries and cakes, shortbread, biscuits, dry biscuits, toast, ice cream and ices; alcoholic beverages and spirits (brandy, grappa, liqueurs, whiskey) and non-carbonated soft drinks (orange juice, coca-cola, fruit juice, etc.) and beer.

Each diet leaflet included 28 pages. On the first page there were the: introduction, instructions for completing the food diary and a detailed list of recommended foods. The subsequent pages were used to record the daily diet for the period of study and thus allow assessment of the level of compliance with the recommended diet.

## References

[1] Isomaa B., Almgren P., Tuomi T., Forsén B., Lahti K., Nissén M., Taskinen M.R., Groop L. (2001). Cardiovascular morbidity and mortality associated with the metabolic syndrome. Diabetes Care.

[2] Schmidt C., Bergström G.M. (2012). The metabolic syndrome predicts cardiovascular events: Results of a 13-year follow-up in initially healthy 58-year-old men. Metab. Syndr. Relat. Disord..

[3] Athyros V.G., Ganotakis E.S., Elisaf M., Mikhailidis D.P. (2005). The prevalence of the metabolic syndrome using the National Cholesterol Educational Program and International Diabetes Federation definitions. Curr. Med. Res. Opin..

[4] Ford E.S., Li C., Zhao G. (2010). Prevalence and correlates of metabolic syndrome based on a harmonious definition among adults in the US. J. Diabetes.

[5] Beltrán-Sánchez H., Harhay M.O., Harhay M.M., McElligott S. (2013). Prevalence and trends of metabolic syndrome in the adult U.S. population, 1999–2010. J. Am. Coll. Cardiol..

[6] Riediger N.D., Clara I. (2011). Prevalence of metabolic syndrome in the Canadian adult population. CMAJ.

[7] Yamaoka K., Tango T. (2012). Effects of lifestyle modification on metabolic syndrome: A systematic review and meta-analysis. BMC Med..

[8] Akbaraly T.N., Singh-Manoux A., Tabak A.G., Jokela M., Virtanen M., Ferrie J.E., Marmot M.G., Shipley M.J., Kivimaki M. (2010). Overall diet history and reversibility of the metabolic syndrome over 5 years: The Whitehall II prospective cohort study. Diabetes Care.

[9] Case C.C., Jones P.H., Nelson K., O’Brian Smith E., Ballantyne C.M. (2002). Impact of weight loss on the metabolic syndrome. Diabetes Obes. Metab..

[10] Kastorini C.-M., Milionis H.J., Esposito K., Giugliano D., Goudevenos J.A., Panagiotakos D.B. (2011). The effect of Mediterranean diet on metabolic syndrome and its components: A meta-analysis of 50 studies and 534,906 individuals. J. Am. Coll. Cardiol..

[11] Estruch R., Ros E., Salas-Salvadó J., Covas M.I., Corella D., Arós F., Gómez-Gracia E., Ruiz-Gutiérrez V., Fiol M., Lapetra J. (2013). Primary prevention of cardiovascular disease with a Mediterranean diet. N. Engl. J. Med..

[12] Salas-Salvadó J., Bulló M., Babio N., Martínez-González M.Á., Ibarrola-Jurado N., Basora J., Estruch R., Covas M.I., Corella D., Arós F. (2011). Reduction in the incidence of type 2 diabetes with the Mediterranean diet: Results of the PREDIMED-Reus nutrition intervention randomized trial. Diabetes Care.

[13] Estruch R. (2010). Anti-inflammatory effects of the Mediterranean diet: The experience of the PREDIMED study. Proc. Nutr. Soc..

[14] Paniagua J.A. (2016). Nutrition, insulin resistance and dysfunctional adipose tissue determine the different components of metabolic syndrome. World J. Diabetes.

[15] Alwahsh S.M., Gebhardt R. (2017). Dietary fructose as a risk factor for non-alcoholic fatty liver disease (NAFLD). Arch. Toxicol..

[16] Jurca R., Lamonte M.J., Barlow C.E., Kampert J.B., Church T.S., Blair S.N. (2005). Association of muscular strength with incidence of metabolic syndrome in men. Med. Sci. Sports Exerc..

[17] Pedersen B.K., Akerström T.C., Nielsen A.R., Fischer C.P. (2007). Role of myokines in exercise and metabolism. J. Appl. Physiol..

[18] Boström P., Wu J., Jedrychowski M.P., Korde A., Ye L., Lo J.C., Rasbach K.A., Boström E.A., Choi J.H., Long J.Z. (2012). A PGC1-α-dependent myokine that drives brown-fat-like development of white fat and thermogenesis. Nature.

[19] Colaianni G., Cuscito C., Mongelli T., Pignataro P., Buccoliero C., Liu P., Lu P., Sartini L., Di Comite M., Mori G. (2015). The myokine Irisin increases cortical bone mass. Proc. Natl. Acad. Sci. USA.

[20] Reza M.M., Subramaniyam N., Sim C.M., Ge X., Sathiakumar D., McFarlane C., Sharma M., Kambadur R. (2017). Irisin is a pro-myogenic factor that induces skeletal muscle hypertrophy and rescues denervation-induced atrophy. Nat. Commun..

[21] Xiong X.Q., Chen D., Sun H.J., Ding L., Wang J.J., Chen Q., Li Y.H., Zhou Y.B., Han Y., Zhang Y.M. (2015). FNDC5 overexpression and Irisin ameliorate glucose/lipid metabolic derangements and enhance lipolysis in obesity. Biochim. Biophys. Acta.

[22] Benedini S., Dozio E., Invernizzi P.L., Vianello E., Banfi G., Terruzzi I., Luzi L., Corsi Romanelli M.M. (2017). Irisin: A Potential Link between Physical Exercise and Metabolism-An Observational Study in Differently Trained Subjects, from Elite Athletes to Sedentary People. J. Diabetes Res..

[23] ClinicalTrials.gov is a database of privately and publicly funded clinical studies conducted around the world. https://www.clinicaltrials.gov/.

[24] Expert Panel on Detection, Evaluation, and Treatment of High Blood Cholesterol in Adults (2001). Executive summary of the third report of the national cholesterol education program (NCEP) expert panel on detection, evaluation, and treatment of high blood cholesterol in adults (adult treatment panel III). JAMA.

[25] Trichopoulou A., Martínez-González M.A., Tong T.Y., Forouhi N.G., Khandelwal S., Prabhakaran D., Mozaffarian D., de Lorgeril M. (2014). Definitions and potential health benefits of the Mediterranean diet: Views from experts around the world. BMC Med..

[26] Elia A. (2010). La Rivoluzione Degli Integrali Buoni (Senza L’Aggiunta di Crusca o Fibre) (The Revolution of Good Whole Grains: Without Bran and Fibers Addition).

[27] Choi H.Y., Kim S., Park J.W., Lee N.S., Hwang S.Y., Huh J.Y., Hong H.C., Yoo H.J., Baik S.H., Youn B.S. (2014). Implication of circulating Irisin levels with brown adipose tissue and sarcopenia in humans. J. Clin. Endocrinol. Metab..

[28] Colaianni G., Mongelli T., Cuscito C., Pignataro P., Lippo L., Spiro G., Notarnicola A., Severi I., Passeri G., Mori G. (2017). Irisin prevents and restores bone loss and muscle atrophy in hind-limb suspended mice. Sci. Rep..

[29] Natalicchio A., Marrano N., Biondi G., Spagnuolo R., Labarbuta R., Porreca I., Cignarelli A., Bugliani M., Marchetti P., Perrini S. (2017). The Myokine Irisin Is Released in Response to Saturated Fatty Acids and Promotes Pancreatic Beta-Cell Survival and Insulin Secretion. Diabetes.

[30] Moreno-Navarrete J.M., Ortega F., Serrano M., Guerra E., Pardo G., Tinahones F., Ricart W., Fernandez-Real J.M. (2013). Irisin Is Expressed and Produced by Human Muscle and Adipose Tissue in Association With Obesity and Insulin Resistance. J. Clin. Endocrinol. Metab..

[31] Liu T.Y., Shi C.X., Gao R., Sun H.J., Xiong X.Q., Ding L., Chen Q., Li Y.H., Wang J.J., Kang Y.M. (2015). Irisin inhibits hepatic gluconeogenesis and increases glycogen synthesis via the PI3K/Akt pathway in type 2 diabetic mice and hepatocytes. Clin. Sci..

[32] Choi Y.K., Kim M.K., Bae K.H., Seo H.A., Jeong J.Y., Lee W.K., Kim J.G., Lee I.K., Park K.G. (2013). Serum Irisin levels in new-onset type 2 diabetes. Diabetes Res. Clin. Pract..

[33] Sanchis-Gomar F., Alis R., Pareja-Galeano H., Sola E., Victor V.M., Rocha M., Hernandez-Mijares A., Romagnoli M.L. (2014). Circulating Irisin levels are not correlated with BMI, age, and other biological parameters in obese and diabetic patients. Endocrine.

[34] Timmons J.A., Baar K., Davidsen P.K., Atherton P.J. (2012). Is Irisin a human exercise gene?. Nature.

[35] Quiñones M., Folgueira C., Sanchez-Rebordelo E., Al-Massadi O. (2015). Circulating Irisin Levels Are Not Regulated by Nutritional Status, Obesity, or Leptin Levels in Rodents. Mediat. Inflamm..

[36] Hee P.K., Zaichenko L., Brinkoetter M., Thakkar B., Sahin-Efe A., Joung K.E., Tsoukas M.A., Geladari E.V., Huh J.Y., Dincer F. (2013). Circulating Irisin in relation to insulin resistance and the metabolic syndrome. J. Clin. Endocrinol. Metab..

[37] Yan B., Shi X., Zhang H., Pan L., Ma Z., Liu S., Liu Y., Li X., Yang S., Li Z. (2014). Association of serum Irisin with metabolic syndrome in obese Chinese adults. PLoS ONE.

[38] Suk Shim Y., Jae Kang M., Yang S., Tae Hwang I. (2017). Irisin is a biomarker for metabolic syndrome in prepubertal children. Endocr. J..

[39] Jang H.B., Kim H.J., Kang J.H., Park S.I., Park K.H., Lee H.J. (2017). Association of circulating Irisin levels with metabolic and metabolite profiles of Korean adolescents. Metabolism.

[40] Ko B.J., Park K.H., Shin S., Zaichenko L., Davis C.R., Crowell J.A., Joung H., Mantzoros C.S. (2016). Diet quality and diet patterns in relation to circulating cardiometabolic biomarkers. Clin. Nutr..

[41] Anastasilakis A.D., Polyzos S.A., Saridakis Z.G., Kynigopoulos G., Skouvaklidou E.C., Molyvas D., Vasiloglou M.F., Apostolou A., Karagiozoglou-Lampoudi T., Siopi A. (2014). Circulating Irisin in healthy, young individuals: Day-night rhythm, effects of food intake and exercise, and associations with gender, physical activity, diet, and body composition. J. Clin. Endocrinol. Metab..

[42] Schlögl M., Piaggi P., Votruba S.B., Walter M., Krakoff J., Thearle M.S. (2015). Increased 24-hour ad libitum food intake is associated with lower plasma Irisin concentrations the following morning in adult humans. Appetite.

[43] De la Iglesia R., Lopez-Legarrea P., Crujeiras A.B., Pardo M., Casanueva F.F., Zulet M.A., Martinez J.A. (2014). Plasma Irisin depletion under energy restriction is associated with improvements in lipid profile in metabolic syndrome patients. Clin. Endocrinol..

[44] Grundy S.M., Cleeman J.I., Daniels S.R., Donato K.A., Eckel R.H., Franklin B.A., Gordon D.J., Krauss R.M., Savage P.J., Smith S.C. (2005). Diagnosis and management of the metabolic syndrome: An American Heart Association/National Heart, Lung, and Blood Institute Scientific Statement. Circulation.

[45] Crujeiras A.B., Zulet M.A., Abete I., Amil M., Carreira M.C., Martinez J.A., Casanueva F.F. (2016). Interplay of atherogenic factors, protein intake and betatrophin levels in obese-metabolic syndrome patients treated with hypocaloric diets. Int. J. Obes..

[46] Richter C.K., Skulas-Ray A.C., Champagne C.M., Kris-Etherton P.M. (2015). Plant protein and animal proteins: Do they differentially affect cardiovascular disease risk?. Adv. Nutr..

[47] Srinivas T.R., Ho B., Kang J., Kaplan B. (2015). Post Hoc Analyses after the Facts. Transplantation.

