# Supplementary material for: Irisin Serum Levels in Metabolic Syndrome Patients Treated with Three Different Diets: A Post-Hoc Analysis from a Randomized Controlled Clinical Trial
Source: Nutrients. 2018 Jun 28;10(7):844. doi: 10.3390/nu10070844 (PMC6073260; doi:10.3390/nu10070844)
Supplement: Supplementary file 1 [file nutrients-10-00844-s001.pdf]

Supplementary Materials

Figure S1: Expected [Irisin] (ng/dl) by Vegetable Protein and Saturated Fatty Acids Daily Intake

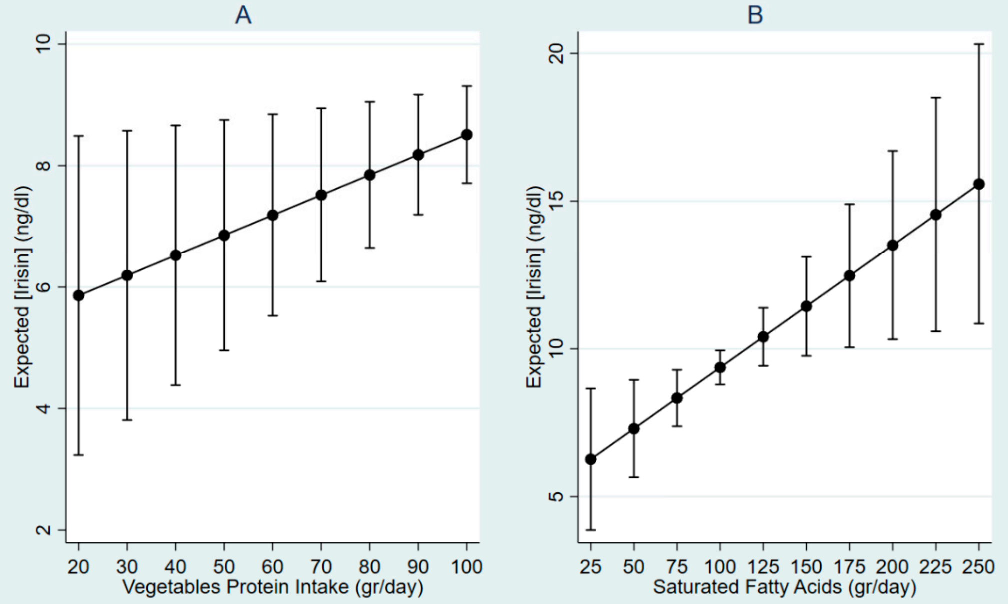

Figure S2: Expected [Irisin] (ng/dl) by Free Fat Mass (%) in LGID

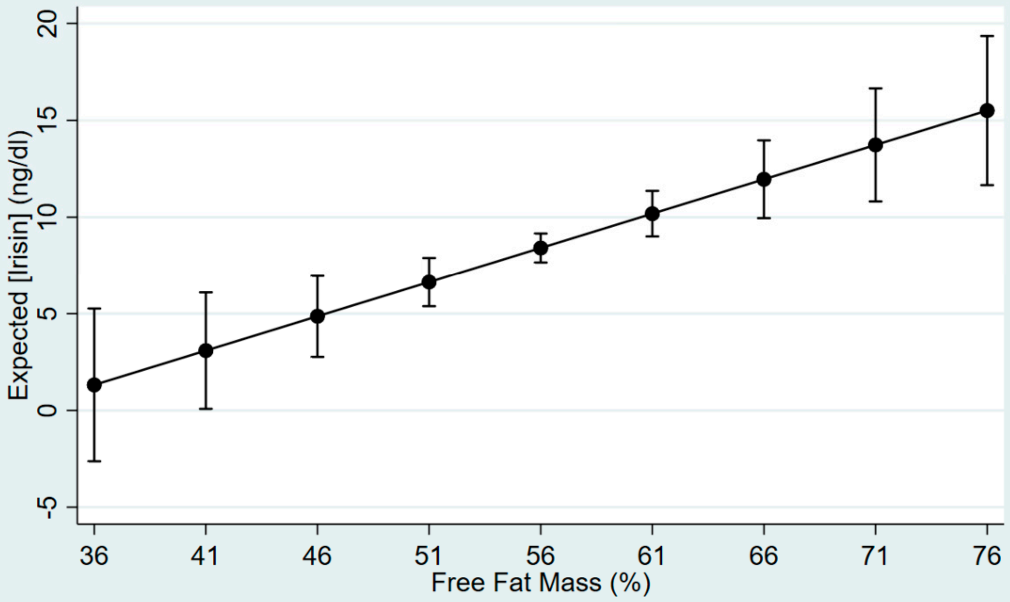

LGID: Low Glycemic Index Diet

Figure S3: Expected [Irisin] (ng/dl) by Body Mass Index in LGIMD

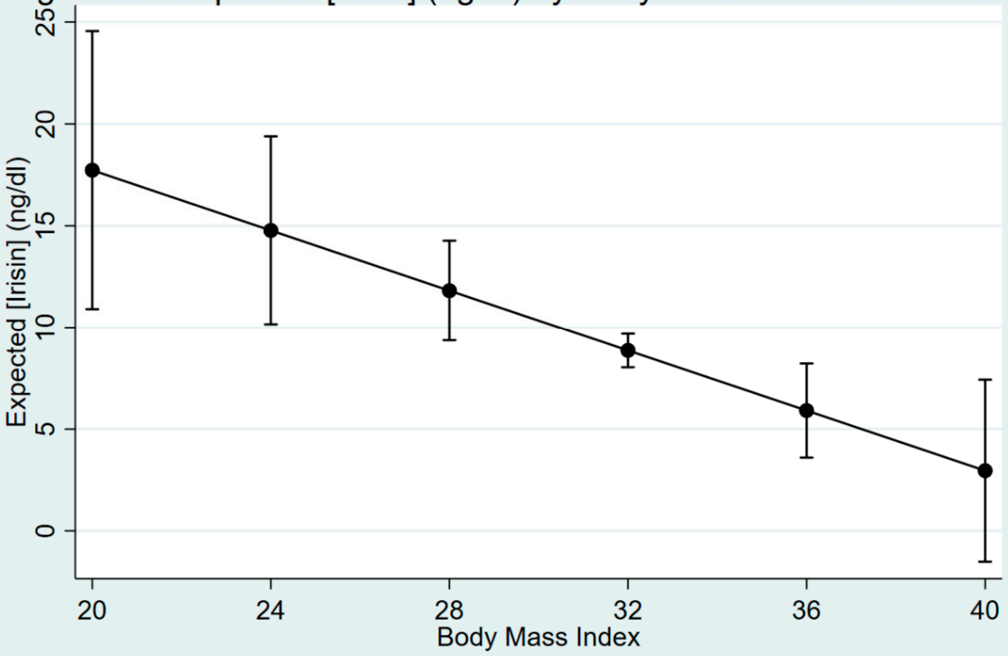

LGIMD: Low Glycemic Index Mediterranean Diet

Table S1: Biochemical and Metabolic Characteristics of Participants

Blood test	Without Diet n=125	LGID n=55	MD n=51	LGIMD n=50
	mean±SD	mean±SD	mean±SD	mean±SD
Glucose (mmol/L)	6.23 ± 1.34	6.87±1.95	6.29±1.18	7.27±2.32
HemoglobinGlycosylated (%)	5.76±0.84	6.19±1.16	5.90±0.71	6.57±1.49
Total Bilirubin (umol/L)	14.54 ± 7.53	17.45±12.83	15.05±4.96	15.39±5.47
Direct Bilirubin (umol/L)	7.70± 31.47	5.47±2.91	5.30±1.71	5.30±1.03
AST (ukat/L)	0.31 ± 0.11	0.24±0.12	0.22±0.06	0.24±0.09
ALT (ukat/L)	0.35 ±0.18	0.37±0.21	0.33±0.16	0.40±0.20
GGT (ukat/L)	0.33 ±0.34	0.37±0.37	0.32±0.22	0.38±0.40
Alkaline Phosphatase	0.89 ± 0.27	0.92±0.33	0.88±0.20	0.94±0.27
Total cholesterol (mmol/L)	4.94 ± 1.04	5.38±1.12	5.43±1.06	5.07±1.11
HDL cholesterol (mmol/L)	1.25 ± 0.31	1.23±0.24	1.22±0.24	1.17±0.34
Triglycerides (mmol/L)	1.40 ±1.00	2.15±1.03	2.16±1.27	2.33±1.27
Homa IR	2.58 ± 2.57	4.38±2.01	4.18±3.72	4.94±3.54
White Blood Cell (x10^9/L)	6.25 ± 1.81	6.53±1.33	6.6±1.67	6.40±1.39
Red Blood Cell (x10^{12}/L)	4.87 ± 0.54	4.81±0.45	4.86±0.40	4.78±0.44
Hemoglobin (g/L)	142.1 ± 14.2	143.0±15.6	147.0±11.3	143.0±15.6
Hematocrit (%)	0.42 ±0.04	0.42±0.03	0.43±0.03	0.42±0.04
Platelet (x10^9/L)	231.18 ± 68.5	224.2±60.1	230.5±50.1	229.3±58.0
Irisina (ng/dl)	17.84 ± 10.9	6.55±5.06	8.12±5.23	8.54±5.45
SCCA-IgM (AU/mL)	83.8±74.3	127.5±167.6	116.6±180.3	139.7±279.7
FMM (Kg)	55.5 ± 11.1	57.85 ± 12.3	58.79 ± 13.3	60.09 ± 12.18
FM (Kg)	23.49 ± 9.76	30.13 ± 13.1	26.61 ± 7.47	28.35 ± 8.35
Phase angle (°)	6.80 ± 5.28	7.19 ± 1.15	7.44 ± 1.20	8.49 ± 9.83

LGID: Low Glycemic Index Diet; MD: Mediterranean Diet; LGIMD: Low Glycemic Index Mediterrranean Diet; SD: Standard Deviation

Table S2: Effect of Biochemical Markers on Irisin Levels.
MEDIDIET, Castellana Grotte, BA, Italy 2009

	Δ[Irisin] ng/dl	95%CI	
Glycemic	-0.00	-0.02	0.02
BT	-0.81	-2.35	0.72
BD	-0.60	-7.30	6.10
GOT	-0.10	-0.29	0.09
GPT	-0.00	-0.10	0.09
GGT	-0.02*	-0.05	-0.00
FA	-0.00	-0.04	0.03
Total Cholesterol	-0.04	-0.42	0.35
HDL-Cholesterol	0.11	-0.29	0.50
LDL-Cholesterol	0.01	-0.37	0.40
Triglycerides	0.00	-0.08	0.08
Apo B	0.02	-0.01	0.04

* $p<0.05$

Table S3: Effect of Food Groups on Irisin Levels
MEDIDIET, Castellana Grotte, BA, Italy 2009

	Δ[Irisin] ng/dl ^	95%CI	
Cheeses(g)	0.00*	0.00	0.00
Processed Meat Products(g)	-0.00*	-0.00	-0.00

* $p<0.05$; ^Adjusted for each other Food Group

Table S4: Effect of BIA on Irisin Levels
MEDIDIET, Castellana Grotte, BA, Italy 2009.

	Δ[Irisin] ng/dl	95%CI	
Free Fat Mass	0.04	-0.03	0.11
Fat Mass	-0.05	-0.11	0.01
Phase Angle	-0.03	-0.13	0.08

Table S5: Descriptive statistics (mean ± standard deviation or relative frequency) of the main characteristics of the subjects with metabolic syndrome (MetS), randomized to Mediterranean diet (MD), low glycaemic index diet (LGID), low glycaemic index Mediterranean diet (LGIMD).

	MD (n=55)	LGID (n=56)	LGIMD (n=53)	p-value
Gender, males (%)	37 (68.5)	32 (57.1)	31 (58.5)	0.41°
Age (years) (M±SD)	58.9±11.0	58.4±10.4	58.4±9.3	0.96*
MetS Score (0-5) (M±SD)	3.5±0.6	3.8±0.7	3.8±0.7	0.06*
Diabetes (%)	6 (10.9)	9 (16.1)	14 (26.4)	0.10°
Hypertension (%)	50 (90.9)	52 (92.9)	50 (94.3)	0.79°
Low Cholesterol HDL (%)	21 (38.9)	25 (44.6)	25 (47.2)	0.67°
Hypertriglyceridemia (%)	33 (60.0)	42 (75.0)	32 (60.4)	0.17°
Dieting at baseline (%)	1 (1.8)	0 (0.0)	1 (1.9)	0.59°

Chi-square test; * ANOVA, F test.

Table S6: Mean and standard deviation (M±SD) of the metabolic syndrome (MetS) score and its components at baseline (T0), and of their variation at 3 (Δ0-3) and 6 months (Δ0-6) in subjects with MetS randomized to Mediterranean diet (MD), low glycaemic index diet (LGID), and low glycaemic index Mediterranean diet (LGIMD).

Variables	Diet	T_0	Δ_{0-3}		Δ_{0-6}	
		M±SD	M±SD	p-value*	M±SD	p-value*
Metabolic Syndrome Score (0-5)	MD	3.5±0.6	1.1±1.2	<0.0001	1.2±1.1	<0.0001
	LGID	3.8±0.7	0.9±1.1	<0.0001	1.0±1.2	<0.0001
	LGIMD	3.8±0.7	0.8±1.2	<0.0001	1.0±1.3	<0.0001
MetS (%)	MD	54/54 (100)	25/54 (46.3)	<0.0001§	26/50 (52.0)	<0.0001§
	LGID	56/56 (100)	41/55 (74.5)	0.0002 §	32/52 (61.5)	<0.0001§
	LGIMD	53/53 (100)	36/53 (67.9)	<0.0001§	27/47 (57.4)	<0.0001§
Waist Circumference (cm)	MD	104.9±9.8	3.3±3.2	<0.0001	2.9±3.5	<0.0001
	LGID	105.4±10.3	3.8±3.0	<0.0001	4.0±4.3	<0.0001
	LGIMD	109.9±12.6	3.2±2.8	<0.0001	3.7±4.2	<0.0001
Fasting Glycemia (mmol/L)	MD	6.28±1.19	0.48±0.59	<0.0001	0.54±0.49	<0.0001
	LGID	7.00±1.89	0.97±1.35	<0.0001	1.04±1.41	<0.0001
	LGIMD	7.21±2.26	0.79±0.95	<0.0001	0.93±1.40	<0.0001
Triglycerides (mmol/L)	MD	2.08±1.28	0.32±1.30	0.0783	0.33±1.35	0.0892
	LGID	2.24±1.00	0.35±0.95	0.0076	0.28±0.65	0.0030
	LGIMD	2.24±1.30	0.37±1.29	0.0419	0.45±1.04	0.0049
HDL Cholesterol (mmol/L)	MD	1.22±0.24	-0.06±0.25	0.0952	-0.05±0.20	0.0916
	LGID	1.21±0.24	-0.04±0.27	0.2182	-0.03±0.20	0.2504
	LGIMD	1.21±0.33	-0.02±0.22	0.5663	-0.05±0.25	0.1740
Systolic Blood Pressure (Hg mm)	MD	135.6±13.1	8.1±14.8	0.0002	10.9±14.3	<0.0001
	LGID	141.0±16.7	12.0±16.1	<0.0001	13.2±17.6	<0.0001
	LGIMD	140.3±16.4	11.1±16.9	<0.0001	12.1±18.0	<0.0001
Diastolic Blood Pressure (Hg mm)	MD	86.4±7.6	9.0±7.1	<0.0001	9.9±7.7	<0.0001
	LGID	86.9±6.5	9.7±654	<0.0001	8.9±7.9	<0.0001
	LGIMD	88.3±7.1	8.5±7.1	<0.0001	10.8±8.1	<0.0001

* t-test for matched pairs; §McNemar Test for matched pairs.

Table S7: Mean and standard deviation (M±SD) of metabolic and anthropometric variables at baseline (T0), and of their variation at 3 (Δ0-3), and 6 months (Δ0-6) in subjects with metabolic syndrome (MetS) randomized to Mediterranean diet (MD), low glycaemic index diet (LGID), and low glycaemic index Mediterranean diet (LGIMD).

Variables	Diet	T_0	Δ_{0-3}		Δ_{0-6}	
		M±SD	M±SD	p-value*	M±SD	p-value*
Weight (kg)	MD	87.7±15.6	4.7±3.0	<0.0001	5.3±3.5	<0.0001
	LGID	87.3±15.2	4.9±2.8	<0.0001	5.6±3.6	<0.0001
	LGIMD	93.3±16.3	5.2±3.2	<0.0001	5.9±4.3	<0.0001
Fat Mass (kg)	MD	26.7±7.3	2.7±4.2	<0.0001	1.8±4.8	0.0147
	LGID	28.6±9.9	3.3±2.7	<0.0001	3.4±2.9	<0.0001
	LGIMD	32.5±11.8	5.0±6.0	<0.0001	4.6±6.5	<0.0001
Free Fat Mass (kg)	MD	59.6±13.4	0.6±4.2	0.3329	1.5±4.7	0.0391
	LGID	57.4±11.7	0.4±2.5	0.2850	0.8±3.0	0.0575
	LGIMD	60.5±13.1	-0.1±3.5	0.7549	1.1±3.1	0.0188
Fatty Liver Score (0-6)	MD	3.2±2.1	1.0±2.0	0.0003	1.0±2.2	0.0010
	LGID	3.4±1.9	1.1±2.3	0.0009	1.4±2.3	<0.0001
	LGIMD	3.6±2.2	1.1±1.9	0.0002	1.3±2.2	<0.0001
ALT (ukat/L)	MD	0.33±0.16	0.08±0.13	<0.0001	0.10±0.13	<0.0001
	LGID	0.38±0.21	0.11±0.16	<0.0001	0.12±0.18	<0.0001
	LGIMD	0.40±0.20	0.12±0.14	<0.0001	0.13±0.17	<0.0001
Cholesterol (mmol/L)	MD	5.35±1.08	0.32±0.70	0.0013	0.28±0.84	0.0201
	LGID	5.41±1.06	0.36±0.74	0.0006	0.30±0.80	0.0092
	LGIMD	5.16±1.02	0.11±0.92	0.4157	0.11±0.82	0.3827
Glycated Hemoglobin (%)	MD	5.9±0.7	0.1±0.3	0.0005	0.2±0.3	<0.0001
	LGID	6.2±1.1	0.3±0.4	<0.0001	0.4±0.7	0.0001
	LGIMD	6.5±1.5	0.5±0.7	<0.0001	0.6±0.8	<0.0001
Insulin (pmol/L)	MD	102.8±71.5	22.9±54.9	0.0033	18.1±60.4	0.0413
	LGID	102.1±43.1	16.7±40.3	0.0031	9.72±97.2	0.4868
	LGIMD	100.0±52.1	14.58±34.7	0.0049	13.2±60.4	0.1390

* t-test for matched pairs

Table S8: Analysis of variance for repeated measures, at baseline (T0), 3 (T3) and 6 (T6) months of the metabolic syndrome (MetS) score and its components in subjects with metabolic syndrome (MetS) randomized to Mediterranean Diet (MD), low glycaemic index diet (LGID), low glycaemic index Mediterranean diet (LGIMD).

		Time			Diets	Time	Diets x Time	Comparisons
Variables	Diet	T_0	T_3	T_6	p-value*	p-value	p-value	
MetS	MD	3.5±0.6	2.4±1.2	2.4±1.1	0.02	<0.001	0.80	MD vs LGID p=0.04
Score (0-5)	LGID	3.8±0.7	2.9±1.1	2.7±1.1				MD vs LGIMD p=0.007
	LGIMD	3.8±0.7	3.0±1.4	2.8±1.4				LGID vs LGIMD p=0.44
WaistCircumference	MD	104.9±9.8	101.7±9.4	101.5±9.3	0.10	<0.001	0.35	MD vs LGID p=0.76
(cm)	LGID	105.4±10.3	101.7±10.0	101.4±10.1				MD vs LGIMD p=0.04
	LGIMD	109.9±12.6	106.6±12.3	105.1±11.2				LGID vs LGIMD p=0.08
Fasting Glycemia	MD	6.28±1.19	5.8±1.27	5.76±1.16	0.02	<0.001	0.09	MD vs LGID p=0.20
(mmol/L)	LGID	7.00±1.89	6.01±0.89	5.93±0.93				MD vs LGIMD p=0.004
	LGIMD	7.22±2.26	6.42±1.69	6.44±1.67				LGID vs LGIMD p=0.09
Triglycerides	MD	2.08±1.28	1.76±0.76	1.83±0.86	0.92	<0.001	0.72	MD vs LGID p=0.70
(mmol/L)	LGID	2.24±1.00	1.87±1.16	1.93±1.03				MD vs LGIMD p=0.74
	LGIMD	2.24±1.30	1.87±1.01	1.84±0.99				LGID vs LGIMD p=0.96
HDL Cholesterol	MD	1.22±0.24	1.27±0.27	1.26±0.26	0.83	0.01	0.90	MD vs LGID p=0.95
(mmol/L)	LGID	1.21±0.24	1.25±0.30	1.25±0.22				MD vs LGIMD p=0.58
	LGIMD	1.21±0.33	1.22±0.27	1.24±0.30				LGID vs LGIMD p=0.62
Systolic Blood	MD	135.6±13.1	127.4±15.3	123.9±15.2	0.11	<0.001	0.79	MD vs LGID p=0.19
Pressure (Hg mm)	LGID	141.0±16.7	128.9±15.8	126.7±15.9				MD vs LGIMD p=0.04
	LGIMD	140.3±16.4	129.1±14.7	129.7±15.7				LGID vs LGIMD p=0.43
Diastolic Blood	MD	86.4±7.6	77.4±7.2	76.0±7.8	0.31	<0.001	0.49	MD vs LGID p=0.44
Pressure (Hg mm)	LGID	86.9±6.5	76.9±8.0	77.8±9.3				MD vs LGIMD p=0.13
	LGIMD	88.3±7.1	79.8±7.5	77.2±8.5				LGID vs LGIMD p=0.43

* ANOVA for repeated measures, F test.

Table S9: Analysis of variance for repeated measures, at baseline (T0), 3 (T3) and 6 (T6) months of metabolic and anthropometric variables in subjects with metabolic syndrome (MetS) randomized to Mediterranean diet (MD), low glycaemic index diet (LGID), low glycaemic index Mediterranean diet (LGIMD).

Variables	Diet	Time T_0	T_3	T_6	Diets p-value*	Time p-value	Diets x Time p-value	Comparisons
Weight	MD	87.7±15.6	83.0±14.6	81.5±15.0	0.38	<0.001	0.69	MD vs LGID p=0.79
(kg)	LGID	87.3±15.2	82.7±14.6	82.1±15.1				MD vs LGIMD p=0.19
	LGIMD	93.3±16.3	88.1±15.3	85.1±13.9				LGID vs LGIMD p=0.28
Fat Mass (kg)	MD	26.7±7.3	24.0±7.2	25.1±7.9	0.35	<0.001	0.02	MD vs LGID p=0.42
	LGID	28.6±9.9	25.5±10.2	25.7±10.0				MD vs LGIMD p=0.15
	LGIMD	32.5±11.8	27.5±9.9	26.7±9.6				LGID vs LGIMD p=0.49
Fat Free Mass	MD	59.6±13.4	59.1±13.2	57.7±12.9	0.62	<0.001	0.67	MD vs LGID p=0.50
(kg)	LGID	57.4±11.7	57.1±11.7	56.4±12.1				MD vs LGIMD p=0.79
	LGIMD	60.5±13.1	60.6±12.4	58.4±11.4				LGID vs LGIMD p=0.34
FattyLiver Score	MD	3.2±2.1	2.2±2.1	2.2±2.3	0.76	<0.001	0.70	MD vs LGID p=0.85
(0-6)	LGID	3.4±1.9	2.3±2.2	2.0±2.3				MD vs LGIMD p=0.47
	LGIMD	3.6±2.2	2.5±2.1	2.2±2.3				LGID vs LGIMD p=0.59
ALT (ukat/L)	MD	0.33±0.16	0.25±0.11	0.23±0.08	0.17	<0.001	0.61	MD vs LGI p=0.19
	LGID	0.38±0.21	0.27±0.11	0.26±0.11				MD vs LGIMD p=0.07
	LGIMD	0.40±0.20	0.28±0.14	0.27±0.16				LGID vs LGIMD p=0.59
Cholesterol	MD	5.34±1.08	5.02±0.82	5.13±0.78	0.79	0.001	0.44	MD vs LGID p=0.99
(mmol/L)	LGID	5.40±1.06	5.04±1.09	5.13±1.12				MD vs LGIMD p=0.55
	LGIMD	5.16±1.07	5.05±0.90	5.05±0.92				LGID vs LGIMD p=0.54
Glycated Hemoglobin	MD	5.9±0.7	5.8±0.6	5.7±0.6	0.02	<0.001	0.04	MD vs LGID p=0.23
(%)	LGID	6.2±1.1	5.9±0.9	5.8±0.6				MD vs LGIM p=0.006
	LGIMD	6.5±1.5	6.1±1.0	6.1±0.9				LGID vs LGIMD p=0.11
Serum Insulin (pmol/L)	MD	106.2±73.9	81.8±35.2	87.5±56.0	0.87	0.03	0.85	MD vs LGID p=0.74
	LGID	105.5±44.5	86.8±44.5	95.4±99.0				MD vs LGIMD p=0.86
	LGIMD	103.3±53.8	88.3±56.0	86.8±81.1				LGDI vs LGIMD p=0.61

* ANOVA for repeated measures, F test

Table S10: Multiple linear regression models of Fat mass and Glycated Hemoglobin on Mediterranean diet (MD), low glycaemic index diet (LGID), and low glycaemic index +Mediterranean diet (LGIMD) at 3 and 6 months, controlling for age, gender, and baseline value of each variable (MD is the comparison diet).

	LGID (T_3)			LGIMD (T_3)			LGID(T_6)			LGIMD (T_6)		
	β	se(β)	p-value	β	se(β)	p-value	β	se(β)	p-value	β	se(β)	p-value
Fat Mass (kg)	0.48	0.80	0.55	1.40	0.83	0.09	1.34	0.93	0.15	1.98	0.96	0.04
Glycated Hemoglobin (%)	0.09	0.06	0.14	0.14	0.06	0.03	0.05	0.07	0.50	0.01	0.07	0.84

Table S11: Energy and nutrients intake in Mediterranean diet (MD), low glycaemic index diet (LGID), and low glycaemic index Mediterranean diet (LGIMD).

	DIETS *		
	MD	LGID	LGIMD
Caloric intake (Kcal/die)	1532	1537	1521
Proteins (%)	15.9	18.2	16.7
Fats (%)	37.3	33.3	41.8
Carbohydrates (%)	45.4	46.5	39.7
Starch (g/d)	111	62	45
Fibers (g/d)	18,5	24,0	23,2
Alcohol (g/d)	9,9	6,9	9,2
Monounsaturated fatty acids (g/d)	33,3	32,0	37,1
Polyunsaturated fatty acids (g/d)	8,3	7,4	8,8
